# Sex differences in subjective age-associated changes in sleep: a prospective elderly cohort study

**DOI:** 10.18632/aging.104016

**Published:** 2020-11-07

**Authors:** Seung Wan Suh, Ji Won Han, Ji Hyun Han, Jong Bin Bae, Woori Moon, Hye Sung Kim, Dae Jong Oh, Kyung Phil Kwak, Bong Jo Kim, Shin Gyeom Kim, Jeong Lan Kim, Tae Hui Kim, Seung-Ho Ryu, Seok Woo Moon, Joon Hyuk Park, Seonjeong Byun, Jiyeong Seo, Jong Chul Youn, Dong Young Lee, Dong Woo Lee, Seok Bum Lee, Jung Jae Lee, Jin Hyeong Jhoo, Ki Woong Kim

**Affiliations:** 1 Department of Psychiatry, Kangdong Sacred Heart Hospital, Hallym University College of Medicine, Seoul, Korea; 2 Department of Neuropsychiatry, Seoul National University Bundang Hospital, Seongnam, Korea; 3 Department of Psychiatry, Dongguk University Gyeongju Hospital, Gyeongju, Korea; 4 Department of Psychiatry, Gyeongsang National University School of Medicine, Jinju, Korea; 5 Department of Neuropsychiatry, Soonchunhyang University Bucheon Hospital, Bucheon, Korea; 6 Department of Psychiatry, School of Medicine, Chungnam National University, Daejeon, Korea; 7 Department of Psychiatry, Yonsei University Wonju Severance Christian Hospital, Wonju, Korea; 8 Department of Psychiatry, School of Medicine, Konkuk University, Konkuk University Medical Center, Seoul, Korea; 9 Department of Psychiatry, School of Medicine, Konkuk University, Konkuk University Chungju Hospital, Chungju, Korea; 10 Department of Neuropsychiatry, Jeju National University Hospital, Jeju, Korea; 11 Department of Psychiatry, Gyeongsang National University Changwon Hospital, Changwon, Korea; 12 Department of Neuropsychiatry, Kyunggi Provincial Hospital for the Elderly, Yongin, Korea; 13 Department of Neuropsychiatry, Seoul National University Hospital, Seoul, Korea; 14 Department of Psychiatry, Seoul National University, College of Medicine, Seoul, Korea; 15 Department of Neuropsychiatry, Inje University Sanggye Paik Hospital, Seoul, Korea; 16 Department of Psychiatry, Dankook University Hospital, Cheonan, Korea; 17 Department of Psychiatry, Kangwon National University, School of Medicine, Chuncheon, Korea; 18 Department of Brain and Cognitive Sciences, Seoul National University, College of Natural Sciences, Seoul, Korea

**Keywords:** sex characteristics, aging, longitudinal studies, self-report, normative

## Abstract

Subjective age-associated changes in sleep (AACS) and sex differences in AACS have never been prospectively investigated in elderly populations. We compared the AACS every 2 years over a total of 6 years between 4,686 community-dwelling healthy men and women aged 60 years or older who participated in the Korean Longitudinal Study on Cognitive Aging and Dementia. Sleep parameters including sleep duration, latency, and efficiency, mid-sleep time, daytime dysfunction, and overall subjective sleep quality were measured using the Pittsburgh Sleep Quality Index at baseline and at each follow-up. The effects of time and sex on subjective sleep parameters were analyzed using linear mixed-effects models. During the 6 years of follow-up, we observed that overall, sleep latency increased, while daytime dysfunction and sleep quality worsened. Significant sex differences in AACS was found, with women showing shortened sleep duration, delayed mid-sleep time, and decreased sleep efficiency over 6 years. Sleep quality worsened in both groups but a more pronounced change was observed in women. Clinicians should be cautious in determining when to treat declared sleep disturbances in this population.

## INTRODUCTION

Evidence has suggested that the normal aging process involves a wide range of physiological changes, among which impairment in the initiation and maintenance of sleep in older age is one of the most pervasive [1]. For example, a seminal meta-analysis based on objective measures reported that, in healthy individuals aged 60 years or older, sleep efficiency continued to decrease with aging while changes in sleep latency and total sleep time were not significant [2]. These age-associated changes in sleep (AACS) in healthy older adults, based on both subjective and objective measures, have been investigated by a host of researchers over the past several decades (Supplementary Table 1) providing evidence for clinical care guidelines.

As for the methods used to obtain measurements of sleep, previous literature suggested that subjective reports could be biased by personality [3], mood, or memory [4]. However, it has also been proposed that subjective measures might reflect physiological characteristics or internal factors that are fundamentally distinct from objective findings [5, 6] and have their own clinical significance. Additionally, the self-perception of sleep habits differs by sex, with women reporting more frequent sleep disturbances [7], and a much-increased sleep latency [8], compared with men.

However, most studies on subjective AACS were cross-sectional. Since rapidly changing sociocultural factors, such as gender roles, influence sleep considerably [9, 10], cross-sectional comparisons of sleep between different age groups may be biased by cohort effects [11] and may not reliably capture intraindividual AACS. Furthermore, AACS has barely been prospectively investigated in older populations. Although there have been several prospective studies on AACS, they examined adolescents or individuals under 70 years of age [12–14], were limited to the assessment of sleep duration, efficiency, or the frequency of sleep disturbances [12–15], and showed a high number of missing data with non-random dropouts [15]. Moreover, no study has thus far focused on sex differences in subjective AACS in the elderly using a longitudinal design.

In this study, we prospectively investigated a large, nationwide, randomly-sampled, community-dwelling elderly population without major psychiatric or neurological disorders to examine the sex difference in subjective AACS.

## RESULTS

Supplementary Figure 1 shows the flow of study participants. We had 4,686 individuals at baseline after excluding those with significant psychiatric or neurological disorders, of whom 2,248 completed the 6-year follow-up. Participant characteristics at baseline are presented by sex (Table 1) and at eave assessment wave (Supplementary Table 2). Men were younger, more educated, more likely to be employed, less likely to be socioeconomically disadvantaged and to live alone, consumed more alcohol, cigarettes, and coffee, were less depressive, more physically active, more likely to be ill, and less likely to be diagnosed with mild cognitive impairment (MCI) than women at baseline. The mean (SD) follow-up duration of participants was 3.87 (2.35) years. During this period, 600 (12.8 %) participants reported having taken sleeping pills at least once. Compared with those who were lost at any follow-up assessment, participants who completed all four waves were younger (mean age [SD]; 68.52 [5.68] vs. 70.98 [7.02], *p* < 0.001), more educated (mean years of education [SD]; 9.16 [5.19] vs. 7.94 [5.31], *p* < 0.001), less likely to live in rural areas (22.8 % vs. 28.4 %, *p* < 0.001), less likely to live alone (11.6 % vs. 14.3 %, *p* = 0.005), less depressive (mean Geriatric Depression Scale [GDS] score [SD]; 7.14 [4.16] vs. 7.39 [4.02], *p* = 0.044), more physically active (total energy expenditure in kilocalories per week over the last year [SD]; 82.73 [156.59] vs. 68.70 [142.14], *p* = 0.001), and less likely to be diagnosed with MCI (20.9 % vs. 29.9 %, *p* < 0.001). There were no observable differences between the groups in terms of sex ratio, employment status, socioeconomic status, the average amount of alcohol, cigarettes, and coffee consumed, Pittsburgh Sleep Quality Index (PSQI) score, and Cumulative Illness Rating Scale (CIRS) total score.

**Table 1:** Baseline characteristics of the study participants.

	**Men (N = 2,148)**	**Women (N = 2,538)**	***p*^a^**
Age, year	69.39 (6.31)	69.99 (6.61)	0.002
Education, year	10.76 (4.89)	6.71 (4.88)	**<0.001**
Employed (%)	1,011 (47.1)	512 (20.2)	**<0.001**
Low SES (%)^a^	44 (2.1)	99 (3.9)	**<0.001**
Living in a rural area (%)	546 (25.5)	643 (25.5)	0.985
Living alone (%)	105 (4.9)	496 (19.6)	**<0.001**
Alcohol, SU/week^b^	7.88 (16.04)	0.60 (6.50)	**<0.001**
Smoking, packs/day^b^	0.18 (0.78)	0.01 (0.11)	**<0.001**
Coffee, cups/day^b^	1.66 (1.98)	0.95 (1.12)	**<0.001**
GDS, score	6.75 (4.03)	7.70 (4.10)	**<0.001**
Physical activity, kcal/week^b^	108.62 (174.65)	48.24 (118.36)	**<0.001**
CIRS total score	4.44 (2.84)	4.11 (2.60)	**<0.001**
Diagnosed with MCI (%)	486 (22.6)	700 (27.6)	**<0.001**

Values are mean (standard deviation) unless specified otherwise.

^a^ Student *t*-test for continuous variables and *χ*^2^ test for categorical variables.

^b^ Amount averaged over the past one year.

Abbreviations: CIRS, Cumulative Illness Rating Scale; GDS, Geriatric Depression Scale; MCI, mild cognitive impairment; SU, standard unit.

**Figure 1:**
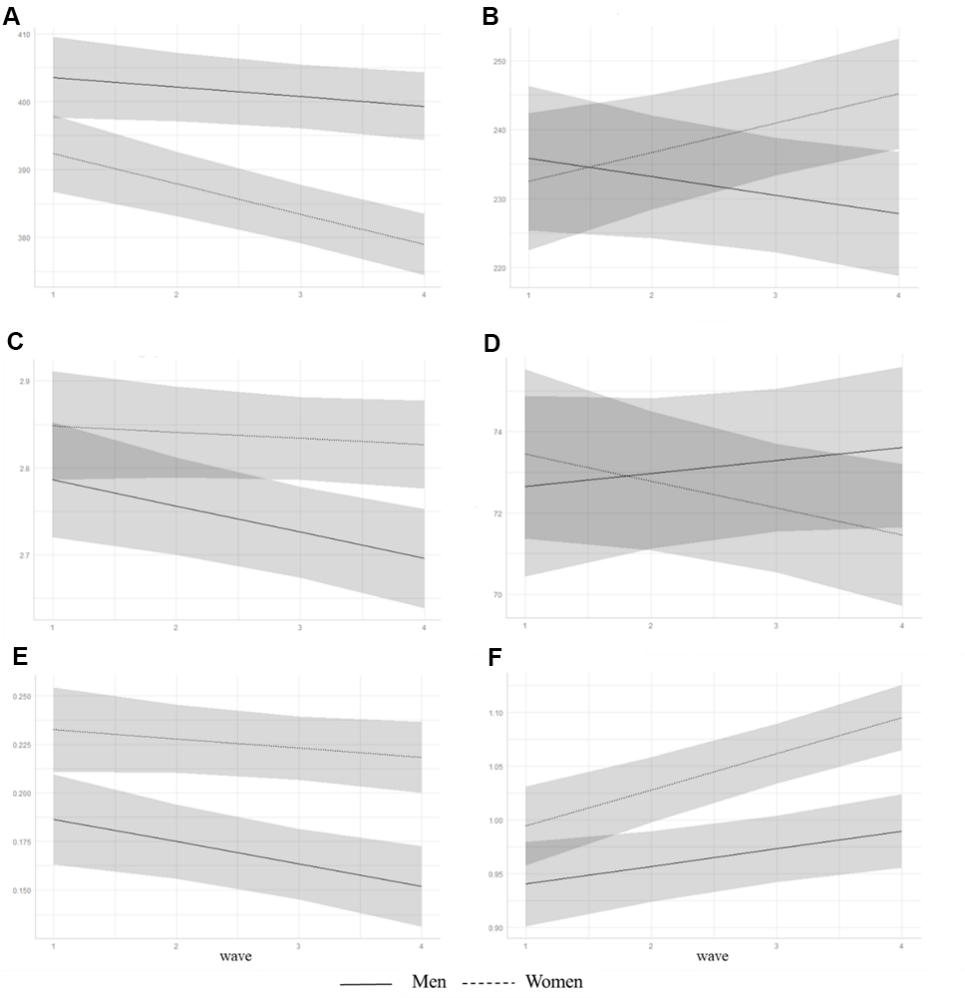
**Trajectories of predicted subjective sleep measures from adjusted linear mixed-effects models for men and women.** Predicted values of (**A**) sleep duration, min; (**B**) mid-sleep time, min; (**C**) log_e_ transformed sleep latency, min; (**D**) sleep efficiency, %; (**E**) log_e_ transformed daytime dysfunction, points; and (**F**) sleep quality, points. Shaded area represents 95% confidence intervals.

Linear mixed-effects models for sleep measures obtained from the PSQI showed that, overall, participants’ sleep latency increased, and daytime dysfunction and sleep quality worsened over 6 years in both the unadjusted and adjusted models. In the adjusted model, women showed shorter sleep duration and more severe daytime dysfunction than men (Table 2, Figure 1).

**Table 2:** Unadjusted and adjusted coefficients for sleep measures using linear mixed-effects models.

**Variable**	**Unadjusted**		**Adjusted^a^**	
	**Coefficient (95% CI)**	***p***	**Coefficient (95% CI)**	***p***
Sleep duration				
Intercept	396.70 (392.67 to 400.72)	**<0.001**	375.32 (340.40 to 410.25)	**<0.001**
Time	-0.55 (-1.96 to 0.86)	0.442	-1.42 (-3.21 to 0.37)	0.121
Sex	-5.13 (-10.59 to 0.33)	0.066	-8.20 (-14.65 to -1.74)	**0.013**
Time * Sex	-3.20 (-5.11 to -1.30)	**0.001**	-3.04 (-5.00 to -1.08)	**0.002**
Mid-sleep time				
Intercept	236.66 (229.41 to 243.90)	**<0.001**	301.83 (240.27 to 363.33)	**<0.001**
Time	-4.14 (-6.72 to -1.56)	**0.002**	-2.67 (-5.93 to 0.59)	0.109
Sex	-21.14 (-30.96 to -11.31)	**<0.001**	-10.23 (-21.89 to 1.44)	0.086
Time * Sex	6.67 (3.18 to 10.15)	**<0.001**	6.90 (3.26 to 10.54)	**<0.001**
Sleep latency^b^				
Intercept	2.79 (2.74 to 2.83)	**<0.001**	2.42 (2.04 to 2.81)	**<0.001**
Time	-0.03^c^ (-0.05 to -0.02)	**<0.001**	-0.03^c^ (-0.05 to -0.01)	**0.005**
Sex	0.15^d^ (0.08 to 0.21)	**<0.001**	0.04 (-0.04 to 0.11)	0.303
Time * Sex	0.02 (-0.002 to 0.04)	0.077	0.02 (-0.001 to 0.05)	0.057
Sleep efficiency				
Intercept	71.33 (69.68 to 72.97)	**<0.001**	66.36 (53.47 to 79.25)	**<0.001**
Time	0.55 (-0.08 to 1.18)	0.088	0.32 (-0.44 to 1.08)	0.410
Sex	2.64 (0.41 to 4.88)	**0.021**	1.77 (-0.88 to 4.42)	0.191
Time * Sex	-1.03 (-1.88 to -0.17)	**0.018**	-0.98 (-1.87 to -0.09)	**0.031**
Daytime dysfunction^b^				
Intercept	0.22 (0.20 to 0.24)	**<0.001**	-0.11 (-0.24 to 0.02)	0.111
Time	-0.01^e^ (-0.02 to -0.004)	**0.003**	-0.01^e^ (-0.02 to -0.003)	**0.006**
Sex	0.05^f^ (0.03 to 0.08)	**<0.001**	0.04^g^ (0.01 to 0.07)	**0.007**
Time * Sex	0.003 (-0.007 to 0.012)	0.557	0.01 (-0.003 to 0.02)	0.182
Sleep quality				
Intercept	0.95 (0.92 to 0.98)	**<0.001**	0.79 (0.56 to 1.02)	**<0.001**
Time	0.02 (0.01 to 0.03)	**0.003**	0.02 (0.003 to 0.03)	**0.013**
Sex	0.08 (0.04 to 0.12)	**<0.001**	0.04 (-0.01 to 0.08)	0.113
Time * Sex	0.01 (-0.001 to 0.028)	0.070	0.02 (0.002 to 0.03)	**0.024**

^a^ Adjusted for age, years of education, employment status, socioeconomic status (whether covered by National Medicaid Program), place of residence (urban vs. rural), presence of cohabitants, physical activity, Geriatric Depression Scale score, amount of alcohol, smoking, and coffee in the past one year, total score of Cumulative Illness Rating Scale, whether diagnosed with mild cognitive impairment, whether being at high risk of obstructive sleep apnea or REM sleep behavior disorder, birth cohort (age < 69 vs. ≥ 69 at baseline), and usage of sleeping pills in the past one month

^b^ Log_e_ transformed; ^c^ +0.97 in minutes; ^d^ +1.16 in minutes; ^e^ +0.99 in points; ^f^ +1.05 in points; ^g^ +1.04 in points

Abbreviations: CI, confidence interval

We also found a significant sex difference in AACS for sleep duration, mid-sleep time, sleep efficiency, and sleep quality under the adjusted model. Post hoc analyses revealed that only women showed decreased sleep duration, delayed mid-sleep time, and decreased sleep efficiency over a period of 6 years (Table 3). Sleep quality worsened in both groups but a more pronounced change was observed in women. The AACS of daytime dysfunction was found only in men with a worsening trend.

**Table 3:** Adjusted coefficients for sleep measures of men and women using linear mixed-effects models.

**Variable**	**Men^a^**		**Women^a^**	
	**Coefficient (95% CI)**	***p***	**Coefficient (95% CI)**	***p***
Sleep duration				
Intercept	350.25 (299.18 to 401.31)	**<0.001**	381.65 (335.33 to 428.01)	**<0.001**
Time	-1.91 (-3.98 to 0.16)	0.072	-4.22 (-6.16 to -2.28)	**<0.001**
Mid-sleep time				
Intercept	345.66 (252.47 to 438.73)	**<0.001**	259.80 (180.52 to 339.06)	**<0.001**
Time	-1.92 (-5.79 to 1.96)	0.333	3.87 (0.46 to 7.28)	**0.026**
Sleep latency^b^				
Intercept	2.67 (2.09 to 3.24)	**<0.001**	2.29 (1.79 to 2.80)	**<0.001**
Time	-0.02^c^ (-0.05 to 0.002)	0.076	-0.01^d^ (-0.03 to 0.01)	0.263
Sleep efficiency				
Intercept	70.65 (51.21 to 90.10)	**<0.001**	6.40 (47.43 to 80.62)	**<0.001**
Time	0.45 (-0.42 to 1.32)	0.309	-0.85 (-1.62 to -0.07)	**0.033**
Daytime dysfunction^b^				
Intercept	-0.38 (-0.68 to -0.08)	**0.012**	0.07 (-0.21 to 0.35)	0.626
Time	-0.02^e^ (-0.03 to -0.01)	**0.007**	-0.004^f^ (-0.02 to 0.01)	0.537
Sleep quality				
Intercept	0.86 (0.52 to 1.19)	**<0.001**	0.81 (0.51 to 1.10)	**<0.001**
Time	0.02 (0.003 to 0.03)	**0.020**	0.03 (0.02 to 0.05)	**<0.001**

^a^ Adjusted for age, years of education, employment status, socioeconomic status (whether covered by National Medicaid Program), place of residence (urban vs. rural), presence of cohabitants, physical activity, Geriatric Depression Scale score, amount of alcohol, smoking, and coffee in the past one year, total score of Cumulative Illness Rating Scale, whether diagnosed with mild cognitive impairment, whether being at high risk of obstructive sleep apnea or REM sleep behavior disorder, birth cohort (age < 69 vs. ≥ 69 at baseline), and usage of sleeping pills in the past one month

^b^ Log_e_ transformed; ^c^ +0.98 in minutes; ^d^ +0.99 in minutes; ^e^ +0.98 in points; ^f^ +1.00 in points

Abbreviations: CI, confidence interval

## DISCUSSION

This study found that community-dwelling healthy elderly Koreans did report changes in subjective sleep habits over time, such that sleep latency increased, and daytime dysfunction and sleep quality worsened over 6 years, while sleep duration, mid-sleep time, and sleep efficiency were largely unchanged. However, we observed significant sex differences in AACS: for every two-year increase in age, women showed a shortening sleep duration by 4.22 minutes, delayed mid-sleep time by 3.87 min, and worsening sleep efficiency by 0.85%. Sleep quality worsened in both men and women by 0.02 and 0.03 points, respectively, with women showing a more statistically pronounced change. In addition, every two years, daytime dysfunction worsened by 0.98 points in men, while no substantial changes were observed in women.

To the best of our knowledge, there have been only a few prospective studies on subjective AACS that included a sizable elderly population. In one study, for every 2 years, weekday sleep duration increased by approximately 15 min whereas weekend sleep duration decreased by approximately 1.5 min compensatorily over 8 years in 8,159 participants aged 57 – 68 years after adjusting for sex and occupation [14]. The researchers suggested that the increase in weekday sleep duration may have been attributed to the retirement of the elderly participants during the follow-up period. However, that study included fairly young elderly adults and the analyses did not adjust for important confounders such as usage of sleeping pills. A recent cohort study with an initial sample size of 6,375 adults aged 42 – 94 years who were followed up to 27 years, reported that sleep efficiency decreased by 3.1% per decade [15]. Though that study accounted for numerous variables such as social class, subjective health rating, marital and working status, and usage of sleeping pills in their linear mixed-effects model, the analysis was not adjusted for cognitive function, as people with MCI could distort the subjective sleep measures [6, 16]. Highly irregular drop-out rates between assessment waves of that study was another limitation that could be a source of bias.

A seminal meta-analysis based on cross-sectional studies of sleep measures by polysomnography or actigraphy suggested that, after 60 years of age, total sleep time decreased non-significantly, sleep latency increased non-significantly, and sleep efficiency decreased significantly, with women having a larger effect size than men [2]. These results were largely in accordance with ours notwithstanding the apparent discrepancy between using self-reported and objective sleep measures.

We also found that, in case of mid-sleep time, men showed a nonsignificant advance while women exhibited a significant delay. These results could be contradictory to the common knowledge that aging is generally characterized by the advance of bedtime and wake-up time to earlier hours [17]. However, a cross-sectional telephone survey conducted in a metropolitan area of France involving 1,026 participants aged 60 and older indicated that the advancement of bedtime and wake-up time was not evident, and even a delaying tendency was observed between women aged 60–64 years and 65–69 years [18]. This phenomenon could be partly explained by the homeostatic effect of sleep need. An increase in sleep need, as shown by pronounced worsening of sleep quality in women, might advance bedtime or delay wake-up time [19] which in turn, coupled with a nonsignificant increase in sleep latency in women as shown in our findings, could lead to a delay in mid-sleep time. It is also possible that the relatively short follow-up period of 6 years could not capture the secular trend of mid-sleep time.

In regard to the self-reported overall sleep quality, which should be distinguished from the global PSQI score that reflects both qualitative and quantitative aspects, previous studies have shown conflicting results in the elderly population. There was a report of a worsening trend of the sleep quality component score from the PSQI in 824 randomly-sampled Japanese elderly participants aged older than 60 years in a cross-sectional study, with women having a more marked change [20], which is in line with our findings. On the other hand, a cross-sectional study from the HypnoLaus Cohort reported that the sleep quality component score from the PSQI improved steadily with age in the 2,966 participants aged between 40 and 80 years old [8], indicating that a spontaneous adaptive adjustment of sleep disturbances might occur in the elderly. However, the latter study excluded approximately 40% of the initial sample of participants who had sleep complaints or any documented sleep disorders, which could have led to a bias toward a super-healthy population.

The underlying mechanisms of the sex differences in AACS or of the individual sleep measure itself are yet to be elucidated. Zhang et al. suggested that this disparity may be attributable to the higher prevalence of depressive mood or anxiety in women compared with men [21]. Though we adjusted our models for depression by including GDS score, it still remains possible that the observed sex difference in AACS is influenced by the presence of affective disorders. Another possible explanation for this phenomenon is the difference between the sexes in the age-associated changes in sex hormones. In older men, sleep fragmentation due to age-associated decrease in testosterone levels could be attenuated by the loss of diurnal fluctuation of the hormone [22]. In contrast, in women, a progressive decrease of estradiol level after menopause may disturb sleep, prolong sleep latency [23], and lead to sleep-disordered breathing through its detrimental effect on the upper respiratory tract [24]. Additionally, women have heightened bodily vigilance and tend to express more somatic symptoms or emotional distress than men [25]. We suggest that it might be the case that subjective AACS concerning sleep duration, mid-sleep time, sleep efficiency, and overall sleep quality might be particularly vulnerable to these effects, though further research is warranted to ascertain these hypotheses.

This study has several limitations. First, the self-reported sleep measures used in our study may lead to a reporting bias related to, as mentioned above, personality, mood, and memory [3, 4]. Nevertheless, there have been reports regarding decent correlations between PSQI and polysomnographic findings in terms of sleep efficiency and latency [26], and between a questionnaire assessing mid-sleep time and sleep duration and corresponding actigraphy findings [27]. Moreover, because self-reported measures are inexpensive and easy to apply, they are highly efficient, and probably the only practical way to collect data over a long-term period with a large sample size. Second, it is possible that 6 years of follow-up was not long enough to capture AACS, leading to false-negative study results. Third, the difference in sleep habits between weekdays and weekends was not taken into account. However, by adjusting for employment status in our analysis models, we believe that we partially compensated for this drawback. Fourth, we did not quantify the duration of naps, which preclude the estimation of sleep duration over a 24-hour period, though instead, we did measure the degree of daytime dysfunction. Fifth, the concept of the “normality” in regard to sleep is difficult to define to date. According to Mowbray et al. [28], the word “normal” can imply several meanings. For practical purposes, we use it in terms of the “statistical” norm where the abnormal is perceived to be that which lies outside the population average range, rather than the “value” norm which takes the ideal, healthier state as its concept. Therefore, we included elderly participants with common sleep problems but excluded those with severe psychiatric or neurologic disorders and with cognitive impairment that could significantly compromise the reliability of the self-reported sleep measures. Sixth, because the participants who completed all follow-ups had substantially different characteristics compared with those who dropped out, with approximately 20% attrition rate per two years, it raised the possibility of bias in our assessment.

In conclusion, for the healthy individuals aged 60 years or older, normative age-associated changes in subjective sleep measures do occur in latency, daytime dysfunction, and sleep quality. As for sex differences, decreased sleep duration, delayed mid-sleep time, and decreased sleep efficiency were found in women, and the worsening of sleep quality was more pronounced in women than in men. It would be imperative for clinicians to understand these changes in sleep habits when determining the necessity to treat declared sleep disturbances of the elderly population.

## MATERIALS AND METHODS

### Participants

This study was conducted as a part of the Korean Longitudinal Study on Cognitive Aging and Dementia (KLOSCAD) [29]. The KLOSCAD is an ongoing nationwide, population-based, prospective elderly cohort study on cognitive aging and dementia. In this study, 6,818 community-dwelling elderly Koreans were randomly sampled from 30 villages and towns across South Korea using residential rosters of the individuals aged 60 years or older. A baseline assessment of the study participants was conducted from November 2010 to October 2012, with follow-ups occurring every two years until the period of November 2017 to October 2018.

To examine the effect of normative human aging, we excluded participants at baseline if they (1) were positive on the Cambridge-Hopkins questionnaire for restless legs syndrome (CHRLSq) [30]; (2) scored 20 or more on Alcohol Use Disorder Identification Test-Korean version (AUDIT-K) [31]; (3) were diagnosed with dementia according to the fourth edition of the Diagnostic and Statistical Manual of Mental Disorders, Text Revision (DSM-IV-TR) [32]; (4) scored 16 or more on the Korean version of Geriatric Depression Scale (GDS-K) [33]; (5) scored 3 or more on the psychiatry category of the CIRS [34]; and (6) scored 3 or more on the neurology category of CIRS. In addition, once an individual was diagnosed with dementia during the study period, we terminated their follow-up and excluded them from that time point, as dementia involves progressive and irreversible neurodegeneration that significantly affects sleep habits [35]. This study was approved by the institutional ethics review board of the Seoul National University Bundang Hospital.

### Assessment of sleep measures

We used the Korean version of the PSQI [36] to obtain subjective sleep measures regarding its duration, mid-sleep time, latency, efficiency, daytime dysfunction, and quality over the past one month at each assessment. We defined mid-sleep time as the midpoint between self-reported sleep onset and wake-up time where sleep onset is the time after sleep latency has elapsed from bedtime [37]. The mid-sleep time reportedly showed excellent agreement with self-awareness chronotype [37] and superior correlation with dim light melatonin onset, the most reliable circadian phase marker in humans, compared with sleep onset or wake-up time [38]. We defined sleep efficiency as the ratio of the self-reported duration of sleep to the time spent in bed and rated daytime dysfunction and sleep quality on a 4-point Likert-type scale with higher scores indicating worsening of symptoms. The “sleep quality” variable, one of the component scores of the PSQI, used in our study denotes the subjective assessment of the overall sleep in a purely qualitative way and was evaluated by asking “How would you rate your sleep quality overall?” This variable needs to be differentiated from the global PSQI score which reflects both the qualitative and quantitative aspects of sleep [39].

### Demographic information and assessment of confounders

Using a study-specific standard interview, trained research nurses collected data on demographic information, physical activity, the amount of alcohol, cigarettes, and coffee consumed over the last one year, and questionnaires including the PSQI, REM Sleep Behavior Disorder Screening Questionnaire (RBDSQ) [40], STOP-Bang [41], CHRLSq, AUDIT-K, GDS-K, and CIRS. We calculated the physical activity over the last one year in terms of total energy expenditure in kilocalories per week, using a formula with relative metabolic rate and metabolic equivalent task as its variables [42]. We quantified the amount of smoking and of alcohol and coffee consumption as packs per day, standard units per week [43], and cups per day, respectively. A score of 5 or more on the RBDSQ indicates a high risk of REM sleep behavior disorder (RBD) [40]. STOP-Bang assesses snoring (S), tiredness during daytime (T), observed apnea (O), high blood pressure (P), body mass index (B), age (A), neck circumference (N), and gender (G), with a score of 5 or more indicating a high risk of obstructive sleep apnea (OSA) [41]. CIRS comprehensively measures the extent and severity of comorbid illnesses on a 5-point scale in regard to the organ-specific categories including cardiovascular, hematopoietic, respiratory, otorhinolaryngologic, gastrointestinal, hepato-renal, genito-urinary, musculoskeletal, neurological, endocrinologic, and psychiatric domains [34].

To assess the cognitive function of study participants, geriatric psychiatrists performed a face-to-face standardized diagnostic test, including physical and neurological examinations, using the Korean version of the Consortium to Establish a Registry for Alzheimer’s Disease Assessment Packet Clinical Assessment Battery (CERAD-K-C) [44] and the Korean version of the Mini International Neuropsychiatric Interview [45]. Trained research neuropsychologists or nurses also performed the CERAD-K Neuropsychological Assessment Battery [44, 46], Digit Span Test [47], and Frontal Assessment Battery [48] on all participants. Results from laboratory tests, such as complete blood cell counts, chemistry panels, apolipoprotein E genotyping, and a serologic test for syphilis, were obtained. A consensus conference attended by four geriatric psychiatrists (KWK, JWH, JHP, and THK) confirmed the final cognitive diagnosis of the participants. Dementia and MCI were diagnosed using the DSM-IV-TR [32] and criteria set by the International Working Group on MCI [49], respectively.

### Statistical analysis

We compared baseline characteristics of study participants between men and women, and between those who completed all four waves of assessment and those who did not using Student *t*-test for continuous variables and χ^2^ test for categorical variables. To analyze the effects of time and sex on subjective sleep measures, six separate linear mixed-effects models were employed, with sleep duration, mid-sleep time, sleep latency, sleep efficiency, daytime dysfunction, and sleep quality as the dependent variables. The effects of time, sex, and their interaction were considered as fixed effects. Intercepts and slopes of individual participants were permitted to vary as random effects.

These models were adjusted for age, years of education, employment status, socioeconomic status (whether covered by National Medicaid Program), place of residence (urban vs. rural), presence of cohabitants, physical activity, GDS-K score, amount of smoking, and alcohol and coffee consumptions in the past one year, CIRS total score, whether diagnosed with MCI, whether at high risk of OSA or RBD, birth cohort (age < 69 vs. ≥ 69 at baseline), and usage of sleeping pills, as these variables have been reported to be associated with age or sex, and related to sleep measures [11, 50–53]. We assumed the missing data over the follow-up to be missing at random. Due to positively skewed distributions, we loge transformed sleep latency, the degree of daytime dysfunction, physical activity, and the amount of smoking, and alcohol and coffee consumptions thereby enhancing the fit of our models. We did not find any apparent heteroscedasticity from the visual inspection of residual plots.

A post hoc analysis for a sleep measure was conducted with men and women separately. The level of significance was set at α = 0.05. Analyses were performed using R Statistical Software (version 3.5.1; R Foundation for Statistical Computing, Vienna, Austria) and the lme4 [54] package.

## Supplementary Material

Supplementary Figure 1

Supplementary Tables
