# Supplementary material for: Sex differences in subjective age-associated changes in sleep: a prospective elderly cohort study
Source: Aging (Albany NY). 2020 Nov 7;12(21):21942–58. doi: 10.18632/aging.104016 (PMC7695390; doi:10.18632/aging.104016)
Supplement: Supplementary Figure 1 [file aging-12-104016-s001..pdf]

SUPPLEMENTARY FIGURE

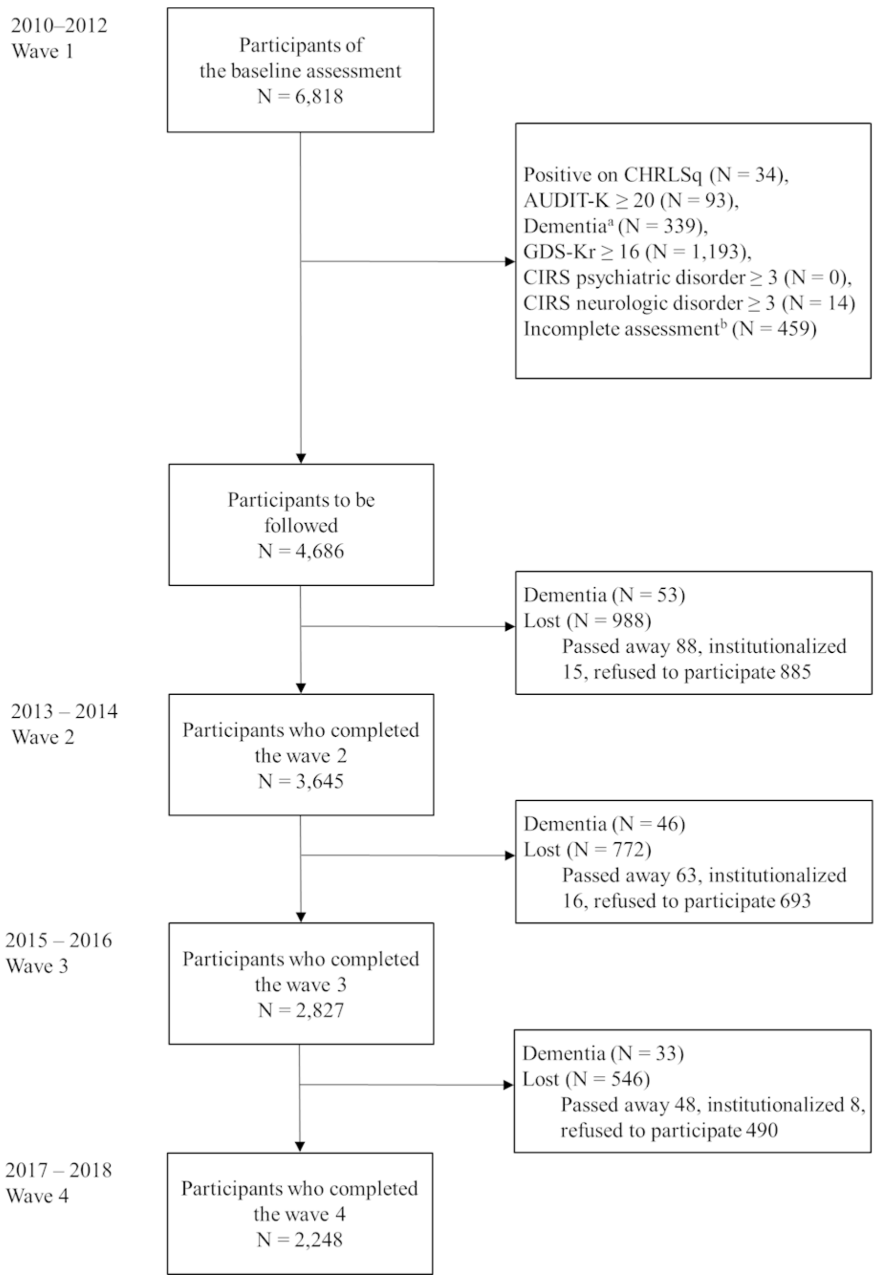

Supplementary Figure 1. Flow chart of the study. [a]Diagnosed according to the fourth edition of the Diagnostic and Statistical Manual of Mental Disorders, Text Revision (DSM-IV-TR) [b]Incomplete assessment of PSQI Abbreviation: PSQI, Pittsburgh Sleep Quality Index; CHRLSq, Cambridge-Hopkins diagnostic questionnaire for restless legs syndrome; AUDIT-K, Alcohol Use Disorders Identification Test – Korean version; GDS-Kr, Korean version of the geriatric depression scale; CIRS, Cumulative Illness Rating Scale.
