# Supplementary material for: Sex differences in subjective age-associated changes in sleep: a prospective elderly cohort study
Source: Aging (Albany NY). 2020 Nov 7;12(21):21942–58. doi: 10.18632/aging.104016 (PMC7695390; doi:10.18632/aging.104016)
Supplement: Supplementary Tables [file aging-12-104016-s002..pdf]

SUPPLEMENTARY TABLES

Supplementary Table 1. Studies investigating age-associated changes in sleep for healthy adults or elderly population.

Study	Setting	n	Age	Women (%)	Assessment	Main Findings	Confounders
Prospective studies							
Didikoglu et al., 2019 [1]	UMLCHA cohort followed for up to 27 years	6,375	42 – 94	69.9	Self-report	SE ↓ (3.1% per decade), SD ↓, average sleep fragmentation ↑, bedtime advanced, getting up time showed minimal change.	Sex, level of education, social class, age, subjective health rating, marital status, working status, smoking, drinking, usage of sleep medication
Akerstedt et al., 2018 [2]	Swedish Longitudinal Occupational Survey of Health (SLOSH) followed for 8 years	8,159	Mean 47.6 (SD 11.6)	56.8	Self-report (Karolinska Sleepiness Scale)	Fatigue decreased across 8 years in all age groups, while sleep problems increased, non-restorative sleep decreased, weekend sleep duration decreased, and weekday sleep duration showed different patterns depending on age.	Gender, occupation
Sforza et al., 2017 [3]	PROOF cohort, followed for 7 years	284	≥ 65	52	PSG	Overall participants: TST ↑<br>Men: TST ↓ (not significant)<br>Women: TST ↑	None
Bliwise et al, 2005 [4]	BASC cohort followed for about 10 years	31	Mean 66.5 (SD 8.0)	67.7	Self-report	Number of nightly awakening ↑, daytime napping ↑, SD →, bed-time →, wake-up time →	Cohabitation status, previous major illnesses
Hoch et al., 1997 [5]	Community-dwelling volunteers followed for 3 years	23 young olds and 27 old olds	Young old: Mean 69.3 (SD 4.0) (Range 61 – 74)<br>Old old: Mean 81.1 (SD 3.5) (Range 75 – 87)	54	**Self-reports** using sleep logs and total score of PSQI, and **PSG**	**Self-report:** None of the diary-based field measures showed effects of time over the 3-year observation period, and none showed group-by-time interactions. No sex by age group by time interactions were significant.<br>**PSG**: % SWS ↓, No age group by time by sex interactions were observed.	Sex, medical burden scores at study entry, changes in medical burden scores over time
Cross-sectional studies							
Ohayon et al., 2004 [6]	Meta-analysis of papers between 1960 and 2003 (65 articles)	3,577	5 – 102	N/A	PSG, actigraphy	Children and adolescents: TST ↓, %SWS ↓, REML ↓, %Stage 2 ↑<br>Adults: TST ↓, SE ↓, %SWS ↓, %REM sleep ↓, REML ↓, SL ↑, %Stage 1 ↑, %Stage 2 ↑, WASO ↑<br>Elderly (≥ 60): SE ↓	
Schwarz et al., 2017 [7]	Sleep and Health in Women Study, population-based, community-	211	Mean 48.9 (SD 11.5) Range 22 – 71	100	Overnight ambulatory PSG	TST ↓, N3 ↓, REM sleep ↓, N1 ↑	

	dwelling						
Conte et al., 2014 [8]	Community-dwelling healthy subjects	20 young adults and 20 elderly	Young adults: 20 - 35; Mean 25.8 (SD 4) / Range 21 – 32 Elderly: ≥ 65; Mean 72.5 (SD 5) / Range 65 – 85	60	PSG	TST ↓, % Stage 2 ↓, SE ↓, SL ↑, % Stage 1 ↑, WASO ↑	None
Morrell et al., 2012 [9]	Wisconsin Sleep Cohort, community-dwelling healthy subjects	ESS, 3,695; MSLT 1,846	Range 30 – 60	46	Self-report (ESS) and MSLT	Male: The association between both subjective and objective sleepiness and SDB diminished significantly with age. Female: No interaction was found between SDB and age.	Comorbidities, depressive symptomology, and BMI
Klerman et al., 2008 [10]	Community-dwelling healthy subjects	35 young adults and 18 elderly	Young adults: Mean 21.9 (SD 3.3) / Range 18-32 / Target 18-30 Elderly: Mean 67.8 (SD 4.3) / Range 60-76/ Target 60-80	45.3	Self-report verified by actigraphy	Increased morning diurnal preference, earlier bedtimes, earlier wake times, no significant difference in mean habitual sleep duration	Absence of sleep disorder
Unruh et al., 2008 [11]	Sleep Heart Health Study (SHHS) cohort	5,407	Men: Mean 63.5 (SD 10.7) Women: Mean 63.6 (SD 11.2)	52	Self-report and PSG	**Self-report** -Men: Not associated with subjective poor sleep quality. -Women: weekend TST ↓, SL ↑, more waking up during the night, waking up too early. **PSG** -Men & women: TST ↓, SE ↓, WASO ↑ -Men: % Stage 1 ↑, % Stage 2 ↑, % SWS ↓, % REM sleep ↓ -Women: not associated with sleep stage.	Race, use of hormone replacement therapy, smoking history, sleep apnea, and chronic health conditions.
Silva et al., 2007 [12]	Sleep Heart Health Study (SHHS) cohort	2,113	≥ 40, Mean 67 (SD 10)	53	Self-report and PSG	**Self-report**: Habitual TST ↓, habitual SL ↑, morning estimated TST ↓, morning estimated SL ↑ **PSG**: TST ↓, SL ↑	Sex, race, BMI, education, time-zone, RDI4%, chronic lung or heart disease, and alcohol or caffeine consumption

Abbreviations: UMLCHA, University of Manchester Longitudinal Study of Cognition in Normal Healthy Old Age; BASC, Bay Area Sleep Cohort; PROOF: PROgnostic indicator OF cardiovascular and cerebrovascular events study; PSQI, Pittsburgh Sleep Quality Index; PSG, polysomnography; ESS, Epworth Sleepiness Scale; MSLT, multiple sleep latency test; SE, sleep efficiency; SD, sleep duration; TST, total sleep time; SWS, slow-wave sleep; REM, rapid eye movement; REML, REM latency; SL, sleep latency; WASO, wake after sleep onset; SDB, sleep-disordered breathing; BMI, body mass index; RDI, respiratory disturbance index
↓, decreased; ↑, increased; →, maintained

Supplementary Table 2. Demographic information and sleep measures at each assessment waves.

Characteristics	Timepoints			
	Wave 1 N = 4,686	Wave 2 N = 3,645	Wave 3 N = 2,827	Wave 4 N = 2,248
Age, year	69.71 (6.48)	71.38 (6.20)	72.82 (5.93)	74.36 (5.66)
Female (%)	2,538 (54.2)	1,982 (54.4)	1,600 (54.5)	1,324 (54.9)
Education, year	8.57 (5.28)	8.90 (5.77)	9.08 (5.40)	9.18 (5.57)
Employed (%)	1,523 (32.5)	1,168 (32.1)	860 (29.3)	681 (28.5)
Low SES (%)[a]	143 (3.1)	93 (2.6)	75 (2.6)	65 (2.7)
Living in rural area (%)	1,189 (25.5)	908 (25.1)	602 (20.5)	393 (16.5)
Living alone (%)	601 (12.9)	522 (14.4)	433 (14.8)	394 (16.5)
Alcohol, SU/week[b]	3.94 (12.41)	3.72 (11.73)	3.04 (9.38)	2.41 (7.39)
Smoking, packs/day[b]	0.09 (0.54)	0.07 (0.29)	0.04 (0.19)	0.04 (0.17)
Coffee, cups/week[b]	1.28 (1.61)	1.29 (1.62)	1.30 (1.32)	1.29 (1.28)
GDS, score	7.26 (4.09)	7.34 (5.31)	7.28 (5.31)	7.27 (5.20)
Physical activity, kcal/week[b]	75.93 (149.91)	65.73 (116.85)	67.10 (126.28)	65.79 (122.51)
CIRS total score	4.26 (2.72)	4.62 (2.71)	5.25 (2.88)	5.90 (3.02)
Diagnosed with MCI (%)	1,186 (25.3)	820 (22.5)	597 (20.3)	436 (18.1)
Amnestic type (% within MCI)	848 (71.5)	516 (62.9)	356 (59.6)	253 (58.0)
Non-amnestic type (% within MCI)	327 (27.6)	284 (34.6)	229 (38.4)	170 (39.0)
Unspecified (% within MCI)	11 (0.9)	20 (2.4)	12 (2.0)	13 (3.0)
High risk of RBD (%)[c]	275 (5.9)	163 (4.5)	116 (4.0)	105 (4.4)
High risk of OSA (%)[d]	363 (8.5)	271 (7.6)	201 (6.9)	150 (6.2)
High risk of RLS (%)[e]	0 (0)	6 (0.2)	7 (0.2)	3 (0.1)
Sleeping pill user (%)	328 (7.0)	248 (6.8)	244 (8.4)	188 (7.8)
Sleep measures				
Men				
Sleep duration, min	396.25 (75.32)	395.10 (74.49)	393.80 (79.28)	394.43 (78.98)
Midsleep time, HH:MM	AM 3:52 (2:20)	AM 3:50 (2:25)	AM 3:44 (2:21)	AM 3:45 (2:22)
Sleep latency, min	21.06 (21.47)	19.52 (20.81)	20.94 (27.52)	20.23 (20.98)
Sleep efficiency, %	71.77 (30.83)	72.61 (31.04)	73.27 (30.51)	72.92 (30.69)
Daytime dysfunction, points	0.32 (0.51)	0.32 (0.53)	0.24 (0.48)	0.30 (0.51)
Sleep quality, points	0.97 (0.56)	0.98 (0.52)	0.98 (0.56)	1.01 (0.60)
Women				
Sleep duration, min	388.83 (79.58)	381.49 (80.14)	378.88 (83.93)	375.48 (84.71)
Midsleep time, HH:MM	AM 3:36 (2:13)	AM 3:42 (2:23)	AM 3:47 (2:25)	AM 3:45 (2:28)
Sleep latency, min	26.21 (27.54)	25.32 (27.10)	25.41 (27.98)	26.61 (27.39)
Sleep efficiency, %	73.47 (30.70)	73.32 (30.91)	72.42 (30.84)	72.10 (31.42)
Daytime dysfunction, points	0.42 (0.57)	0.39 (0.57)	0.36 (0.57)	0.40 (0.55)
Sleep quality, points	1.07 (0.57)	1.08 (0.55)	1.12 (0.60)	1.15 (0.59)

Values are mean (SD) unless specified otherwise.
[a] Covered by the National Medicaid Program. [b] amount averaged over the past 1 year
[c] Scored 5 or higher on REM sleep behavior disorder screening questionnaire
[d] Scored 5 or higher on STOP-Bang questionnaire
[e] Positive on Cambridge-Hopkins questionnaire for restless legs syndrome. SES, socioeconomic status; SU, standard unit; GDS, geriatric depression scale; CIRS, cumulative illness rating scale; MCI, mild cognitive impairment.

Supplementary References

1. Didikoglu A, Maharani A, Tampubolon G, Canal MM, Payton A, Pendleton N. Longitudinal sleep efficiency in the elderly and its association with health. J Sleep Res. 2020; 29:e12898. https://doi.org/10.1111/jsr.12898 PMID:31313420

2. Åkerstedt T, Discacciati A, Miley-Åkerstedt A, Westerlund H. Aging and the change in fatigue and sleep - a longitudinal study across 8 years in three age groups. Front Psychol. 2018; 9:234. https://doi.org/10.3389/fpsyg.2018.00234 PMID:29568279

3. Sforza E, Hupin D, Pichot V, Barthélémy JC, Roche F. A 7-year follow-up study of obstructive sleep apnoea in healthy elderly: the PROOF cohort study. Respirology. 2017; 22:1007–14. https://doi.org/10.1111/resp.13013 PMID:28225159

4. Bliwise DL, Ansari FP, Straight LB, Parker KP. Age changes in timing and 24-hour distribution of self-reported sleep. Am J Geriatr Psychiatry. 2005; 13:1077–82. https://doi.org/10.1176/appi.ajgp.13.12.1077 PMID:16319300

5. Hoch CC, Dew MA, Reynolds CF 3rd, Buysse DJ, Nowell PD, Monk TH, Mazumdar S, Borland MD, Miewald J, Kupfer DJ. Longitudinal changes in diary- and laboratory-based sleep measures in healthy “old old” and “young old” subjects: a three-year follow-up. Sleep. 1997; 20:192–202. https://doi.org/10.1093/sleep/20.3.192 PMID:9178915

6. Ohayon MM, Carskadon MA, Guilleminault C, Vitiello MV. Meta-analysis of quantitative sleep parameters from childhood to old age in healthy individuals: developing normative sleep values across the human lifespan. Sleep. 2004; 27:1255–73. https://doi.org/10.1093/sleep/27.7.1255 PMID:15586779

7. Schwarz JF, Åkerstedt T, Lindberg E, Gruber G, Fischer H, Theorell-Haglöw J. Age affects sleep microstructure more than sleep macrostructure. J Sleep Res. 2017; 26:277–87. https://doi.org/10.1111/jsr.12478 PMID:28093830

8. Conte F, Arzilli C, Errico BM, Giganti F, Iovino D, Ficca G. Sleep measures expressing ‘functional uncertainty’ in elderlies’ sleep. Gerontology. 2014; 60:448–57. https://doi.org/10.1159/000358083 PMID:24732109

9. Morrell MJ, Finn L, McMillan A, Peppard PE. The impact of ageing and sex on the association between sleepiness and sleep disordered breathing. Eur Respir J. 2012; 40:386–93. https://doi.org/10.1183/09031936.00177411 PMID:22241742

10. Klerman EB, Dijk DJ. Age-related reduction in the maximal capacity for sleep—implications for insomnia. Curr Biol. 2008; 18:1118–23. https://doi.org/10.1016/j.cub.2008.06.047 PMID:18656358

11. Unruh ML, Redline S, An MW, Buysse DJ, Nieto FJ, Yeh JL, Newman AB. Subjective and objective sleep quality and aging in the sleep heart health study. J Am Geriatr Soc. 2008; 56:1218–27. https://doi.org/10.1111/j.1532-5415.2008.01755.x PMID:18482295

12. Silva GE, Goodwin JL, Sherrill DL, Arnold JL, Bootzin RR, Smith T, Walsleben JA, Baldwin CM, Quan SF. Relationship between reported and measured sleep times: the sleep heart health study (SHHS). J Clin Sleep Med. 2007; 3:622–30. PMID:17993045
